# Factors affecting the severity and prognosis of visual damage in inhalational methanol poisoning

**DOI:** 10.3389/fmed.2023.1098138

**Published:** 2023-03-22

**Authors:** Hongyi Shen, Liu Xueying Zhong, Yue Fu, Wanwen Shao, Yan Yang, Zhenyu Wang, Hui Yang

**Affiliations:** ^1^Zhongshan Ophthalmic Center, Sun Yat-sen University, Guangzhou, China; ^2^Department of Ophthalmology, The Third Affiliated Hospital of Sun Yat-sen University, Guangzhou, China

**Keywords:** inhalational methanol poisoning, visual function prognosis, optic nerve impairment, risk factors, visual damage

## Abstract

**Background/Aim:**

Inhalational methanol poisoning could cause severe damage to visual function. This study analyzed the prognosis of the correlation between the visual function and the relevant risk factors.

**Methods:**

In this retrospective study, 14 patients had confirmed inhalational methanol poisoning, resulting in visual impairment in both eyes. The following tests were performed: laboratory tests, best corrected visual acuity (BCVA), intraocular pressure, slit lamp, fundus photography, visual field (VF), pattern visual evoked potential (P-VEP), flash electroretinogram (F-ERG), optical coherence tomography (OCT), and orbital or cranial magnetic resonance imaging (MRI).

**Results:**

With active treatment, visual function improved to varying degrees in all 14 cases (28 eyes) in this study. The BCVA of 21 eyes declined to no light perception at the onset; 16 eyes showed different degrees of improvement after treatment, with the final BCVA (LogMar) mainly ranging from 2 to 3, and vision acuity stabilized after the 5th month. The VF pattern in the acute phase was mostly blind. Other VF patterns included the central visual island, large paracentral scotomas, hemipleural VF defects, and the temporal visual island. Retinal nerve fiber layer (RNFL) thickening was observed commonly in the acute phase (146.8 ± 33.2 μm), which subsided in the 1st month, showed dramatic thinning at the 3rd month, and then stabilized in the 5th month after onset. MRI showed that the main sites involved were optic nerve impairment, the optic disk, and retrobulbar segments. The basal ganglia region was the site most involved in the central nervous system (CNS).

**Conclusion:**

Inhalational methanol poisoning could lead to severe impairment of visual function, and the prognosis of visual acuity (VA) was highly relevant to the risk factors of duration of toxic exposure, delayed admission, and degree of acidosis.

## 1. Introduction

Methanol poisoning endangered lives and caused irreversible damage to the visual system. The first reported case dates back to 1923 when the working class accidentally consumed adulterated liquor (1). During that time, most acute cases of methanol poisoning were caused by accidentally consuming methanol-containing products, and children ingested such products unintentionally. By contrast, chronic poisoning from methanol by respiratory inhalation or dermal penetration was rare (2).

In recent years, the incidence of inhalational methanol poisoning has increased with the development of industries in developing countries and the lack of production safety supervision. Repeated inhalation of methanol vapor, in particular, causes chronic accumulation of methanol and its metabolites in the body, eventually reaching the threshold that causes severe optic nerve damage (3).

Current clinical studies on inhalational methanol poisoning lacked a thorough examination of visual function indicators such as VA, VF, and RNFL thickness, as well as the relationship of vision function with the depth of intoxication and systemic conditions. This study presents a detailed retrospective analysis of 14 patients who suffered from inhalational methanol poisoning between 2012 and 2021 and who were all exposed to high concentrations of methanol vapor at work. The related industries included fabric, leather, sunglasses, solid alcohol, cell phone cleaners, and electronics parts processing factories.

Detailed clinical material was evaluated during the acute intoxication period and 6 months of follow-up to better understand the clinical characteristics of chronic inhalational methanol poisoning.

## 2. Materials and methods

### 2.1. Study design and setting

In this retrospective analysis, 14 cases of inhalational methanol poisoning, resulting in visual impairment in both eyes for a total of 28 eyes, were collected. The systemic examinations at the onset of the disease were provided by the local hospital, and all of them underwent a detailed ophthalmic examination in our hospital after transferal to our hospital. All patients were followed up for visual functions within 1 month, 3 months, and 6 months after the onset of the disease.

### 2.2. Selection of participants and treatment protocol

The study was approved by the Ethics Review Committee at the hospital, and all participants signed informed consent for the clinical study. Diagnostic criteria for methanol poisoning include (1) serum methanol >10 mg/dl wt/vol (to convert to millimoles per liter, multiply by 0.0312) and (2) confirmed history of exposure to methanol after investigation and methanol detection failure due to beyond metabolic half-life in patients with at least two of the following: pH <7. 3, serum bicarbonate <20 mEq/l (to convert to millimoles per liter, multiply by 1.0), or osmolal gap >10 mOsm/kg, and exclusion of other diseases which could cause similar clinical symptoms (4).

The treatment protocol was based on the Practice Guidelines on the Treatment of Methanol Poisoning. All patients were treated according to the degree of the disease with acid–base correction in the acute phase. All patients with a pH below 7.3 were treated with sodium bicarbonate and volume expansion with isotonic saline to correct acidosis (5). Furthermore, high-dose glucocorticoid pulse therapy followed by competitive inhibition of methanol oxidation by ethanol and folic acid to aid in the metabolism of formic acid, and neurotrophic and neuroprotective treatments with B vitamins, methylcobalamin, and citicoline in the acute phase and during follow-up are recommended (2). Intermittent or continuous forms of renal dialysis were used in patients with severe intoxication to eliminate formate, methanol, and correct acidemia (5).

### 2.3. Clinical examinations and laboratory tests

At the acute onset, patients underwent laboratory tests, including routine blood, urine, and stool tests, routine biochemistry, coagulation, sedimentation, blood gas analysis, and blood and urine methanol toxicology tests.

All subjects were examined, including BCVA, intraocular pressure, slit lamp, fundus photography, VF, P-VEP, F-ERG, OCT, and optical coherence tomography angiography (OCTA) (Heidelberg, SPECTRALIS HRA + OCT, Germany). Orbital or cranial magnetic resonance imaging (MRI) was scanned at 3.0 T in both the plain and enhancement modes.

A VF examination was performed with a Humphrey visual field analyzer (STATPAC, Allergan Humphrey, San Leandro, CA, United States) using the standard 30–2 procedure with the No.III cursor. The reliability criteria were the following: solid vision loss rate < 20%, false-negative rate < 15%, and false positive rate < 15%; and those who did not meet the criteria were excluded. Retinal nerve fiber layer (RNFL) and retinal ganglion cell layer (GCL) thickness were measured by HD-OCT (Carl Zeiss Meditec, Jena, Germany). Image acquisition was performed by scanning the peripheral 6 × 6 mm area of the optic disk, counting the average RNFL thickness and the thickness of each quadrant of the optic disk (including the superior, inferior, nasal, and temporal sides of the optic disk), and the GCL thickness by scanning the 7 × 7 mm ganglion cell complex layer of the macula, including the macula plexiform layer (mIPL), inner nuclear layer (mINL), and the inner plexiform layer (IPL). Three time points of the disease course, 1 month, 3 months, and 6 months were selected for observation, and changes in the thickness of RNFL and GCL (∆1–0, ∆3–0, and ∆6–0) at each stage of the disease course were statistically analyzed and compared with onset.

### 2.4. Calculations and data analysis

The decimal fraction recorded the BCVA in both eyes and converted it to logMAR BCVA. The conversion formula was based on logMAR = −log (decimal acuity) and recorded as 5, 4, and 3 for no light perception, light perception, and hand movement, respectively (4). The statistical software was IBM SPSS Statistics for Windows, version 19.0 (IBM Corp., Armonk, NY, United States), and statistical significance was set at *p <* 0.05. All data were tested using the Shapiro–Wilk test and conformed to the normal distribution, expressed as mean ± standard deviation (SD). The *t*-test was used to compare groups and one-way ANOVA was used to compare multiple groups. The effect of RNFL thickness on VA prognosis was analyzed using a mixed linear model, and the influence of prognostic VA was analyzed using Spearman’s correlation analysis.

## 3. Results

### 3.1. Participants

A total of 28 eyes in 14 patients with inhalational methanol poisoning with bilateral onset were included. There were 12 male and two female cases, ranging in age from 14 to 70 years, with an average age of 29 (Table 1).

**Table 1:** Basic information for patients with inhalational methanol poisoning.

Patient (No.)	Gender	Age (Years)	Main symptoms	BCVA-baseline (OD)	BCVA-baseline (OS)	BCVA- 6 months (OD)	BCVA- 6 months (OS)
1	Male	14	Painless, binocular vision loss	5	5	2.7	2
2	Female	18	Sudden binocular vision loss	2.2	2.1	3	5
3	Male	27	Headache, binocular vision loss	5	5	2.2	2.9
4	Male	17	Headache, binocular vision loss	5	5	0.3	0.3
5	Female	41	Drowsiness, coma, dyspnea	5	5	2.9	2.7
6	Male	19	Binocular vision loss, dizziness, asthenia	1.5	1.7	0.9	1.5
7	Male	18	Binocular vision loss with painful eye rotation	5	5	2.9	1.7
8	Male	19	Painless, binocular vision loss	4	5	0.6	3
9	Male	29	Vomiting, diarrhea, asthenia	4	4	3	4
10	Male	38	Nausea, chest tightness, shortness of breath, asthenia	5	5	2.1	2.2
11	Male	70	Coma, binocular vision loss	5	5	2.7	2.5
12	Male	46	Blurred binocular vision, dizziness, vomiting	5	5	2.2	0.2
13	Male	33	Intoxicated gait, blurred vision, vomiting	5	5	5	5
14	Male	17	Jet vomiting, headache	5	5	5	5
Mean ± SD	–	29.0 ± 15.0	–	4.4 ± 1.1	4.5 ± 1.1	2.5 ± 1.3	2.7 ± 1.5

VA (LogMar) 0, 0.1, 0.3, 0.5, 0.8, 1.0, and 2.0 were equivalent to VA(Decimal) 1.0, 0.8, 0.5, 0.32, 0.16, 0.1, and 0.01, respectively; VA (LogMar) 3, 4, and 5 were equivalent to VA (Decimal) hand movement, light perception, and no light perception, respectively. BCVA, best corrected visual acuity; VA, visual acuity; ODl, oculus dexter; OS, oculus sinister.

### 3.2. Ophthalmic examination

All subjects underwent a detailed ocular examination at the time of admission. The pupillary changes at the onset were divided into three categories: 18 eyes with dilated pupils, 18 eyes showing a blunt reaction to light, and four with a positive RAPD sign. At the early stage, optic disk edema (17 eyes), retinal edema (13 eyes), retinal venous tortuousness (12 eyes), and dilatation (12 eyes) were found. The optic disk turned pale 2 months gradually after the onset of the disease in 28 eyes and had thinner retinal blood vessels in 18 eyes.

### 3.3. Visual acuity

**Figure 1:**
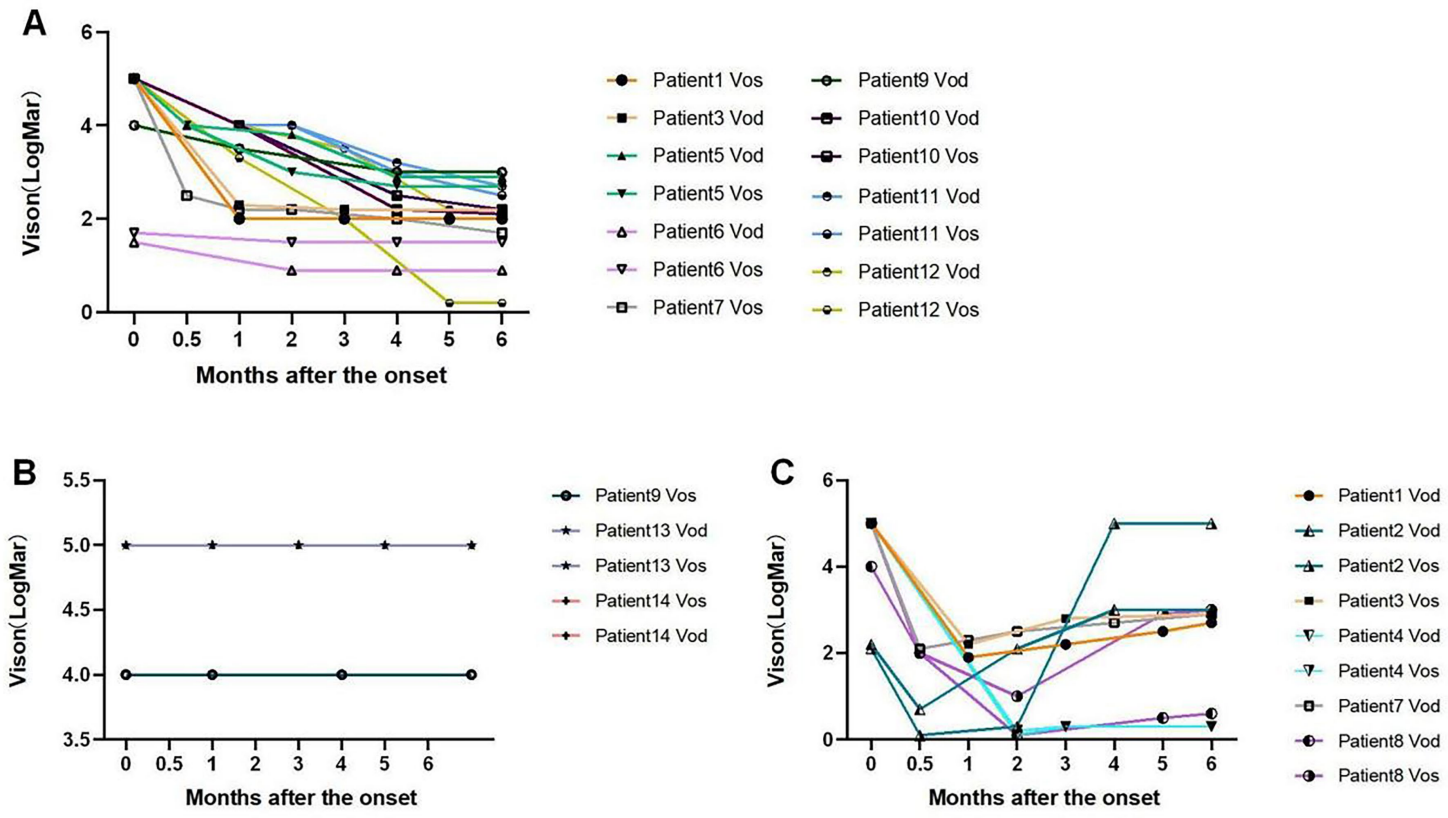
The developing trend of BCVA (logMAR) throughout the patient’s follow-up for 6 months. **(A)** The VA of nine patients (14 eyes) gradually increased after impairment and then remained stable. **(B)** Three patients (5 eyes) had no change in VA after impairment. Patient 9 Vos was in the line of Vision 4.0, Patient 13 Vod, Patient 13 Vos, Patient 14 Vod, and Patient 14 Vos were in the line of Vision 5.0. **(C)** Six patients (9 eyes) had a secondary decrease in VA early after impairment. However, VA turned down after 0.5 to 2 months. Vod, VA of oculus dexter; Vos, VA of oculus sinister.

The BCVA (logMAR) of the patients after the onset of the disease was divided into three main categories (seen in Figure 1). With treatment, the BCVA of 14 eyes showed a gradually increasing trend and became stable after the impairment, with the final BCVA ranging from 0.2 to 3 logMAR. Nine eyes gradually increased and then decreased after impairment, with the final BCVA ranging from 0.3 to 5 logMAR. Five eyes showed no change after impairment, with the final BCVA ranging from 4 to 5 logMAR.

Through analyzing the BCVA curve during the follow-up period of the patients for 6 months, we found that 21 eyes with no light perception recovered their final vision. Of the 17 eyes recovered to LP and above, three eyes recovered to logMAR<1, one eye recovered to 1 ≤ logMAR<2, 12 eyes recovered to 2 ≤ logMAR<3, and the remaining one eye recovered to 3 logMAR (Table 2).

**Table 2:** The visual acuity in 14 cases (28 eyes) in the initial stage and 6 months after the onset.

BCVA (logMAR)	Number of eyes at the beginning of the onset	Number of eyes in the 6 months of the onset
NLP (5 logMAR)	21	5
LP (4 logMAR)	3	1
HM (3 logMAR)	0	3
2 ≤ BCVA(logMAR) < 3	2	12
1 ≤ BCVA(logMAR) < 2	2	2
0 ≤ BCVA(logMAR) < 1	0	5

NLP, no light perception; LP, light perception; HM, hand motion.

### 3.4. Visual field

At the beginning of the disease, most patients were unable to cooperate with VF examinations due to extremely poor VA during the acute poisoning phase. VF examinations were carried out in seven patients during the course of the disease in the 1st month, and the results of the VF examinations are listed later (Table 3).

**Table 3:** VF impairment results in the 1st month after the onset of the disease.

VF impairment manifestations	Number of cases	Percentage (%)
Paracentral scotomas	2	14%
Superior hemianopia defect	1	7%
Tubular VF	1	7%
Total VF loss with relative preservation of the central VF	1	7%
Total loss of VF loss with preserved temporal visual island	1	7%
Total VF loss with preserved nasal view island	1	7%
Total VF defect	7	50%

VF, visual field.

### 3.5. Retinal nerve fiber layer thickness change in 10 cases (20 eyes) underwent optical coherence tomography examination

**Figure 2:**
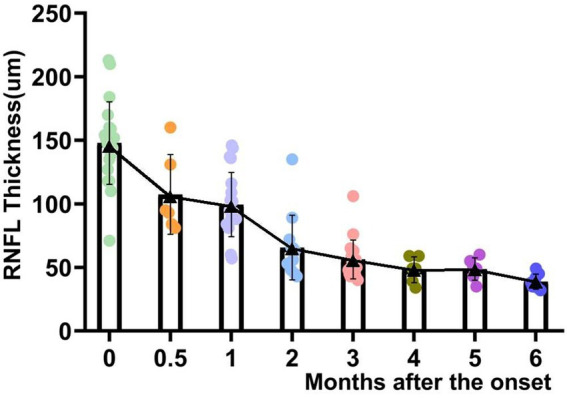
Scattered distribution and changes in the average thickness of RNFL with the course of the disease.

**Figure 3:**
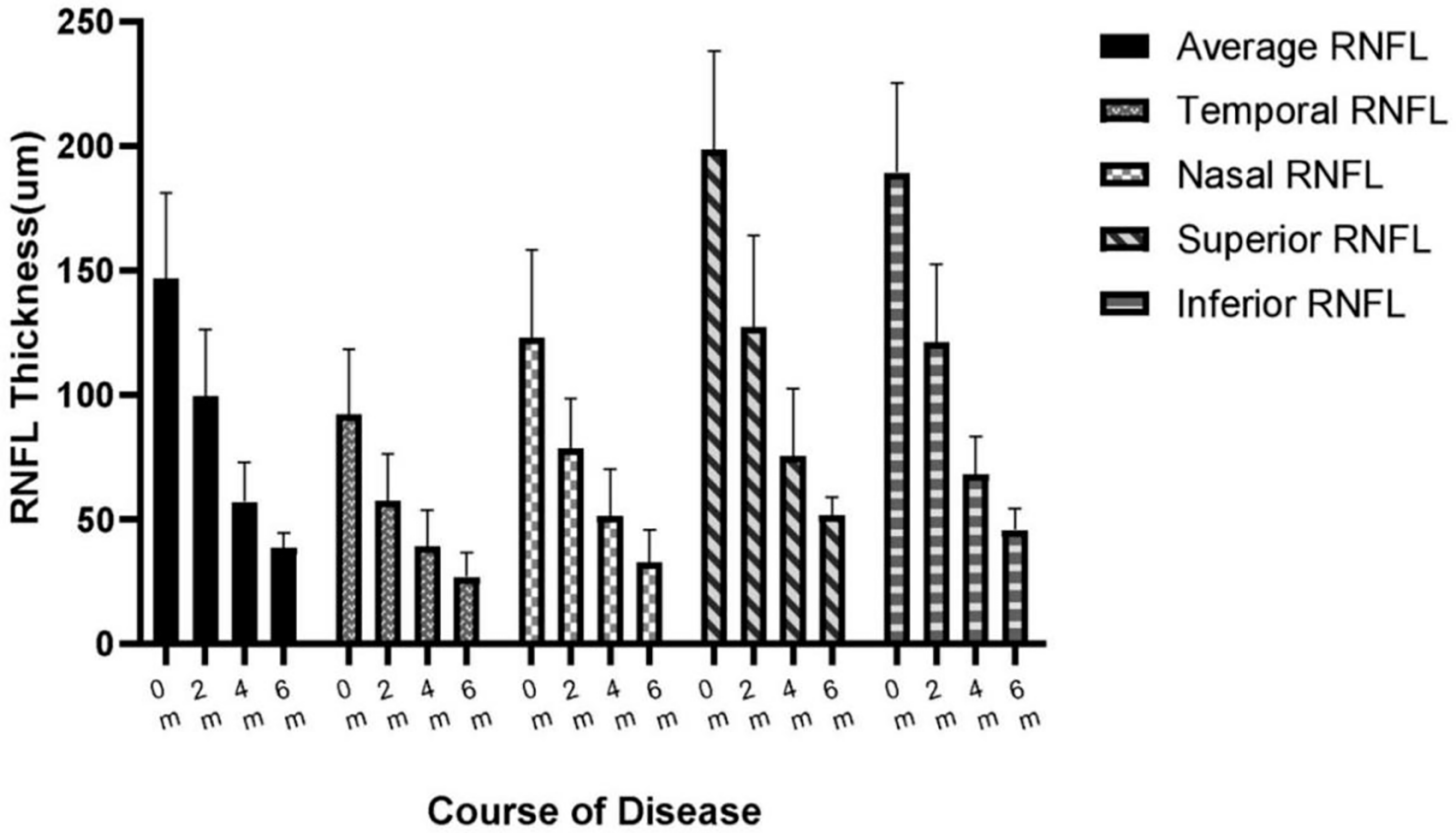
Changes in quadrants and average thickness of RNFL at the onset, 1st, 2nd, 4th, and 6th months of the disease. RNFL, Retinal nerve fiber layer.

The average RNFL thickness of the acute phase of the optic disk was 146.8 ± 33.2 μm at the time of the onset and mostly subsided to 99.7 ± 25.9 μm at the end of the 1st month of the disease. In our study, the RNFL thickness thinning occurred primarily within the first 3 months of the disease to 57.1 ± 15.5 μm. The magnitude of the 3rd- to 6th-months RNFL change slowed down from 57.1 ± 15.5 μm to 38.8 ± 5.6 μm (Figure 2). The percentage change in the thickness of ∆0-1 m RNFL was 32.1%, the thickness of ∆0-3 m RNFL was 61.1%, and the thickness of ∆0-6 m RNFL was 73.6%. The differences between the average RNFL thickness in the 1st, 3rd, and 6th months compared to the onset were statistically significant (*p* < 0.05). The RNFL changes in different quadrants were observed as a pattern of superior > inferior > nasal > temporal thickness thinning can be observed at the onset, 1st, 2nd, 4th, and 6th months of the disease (Figure 3).

**Figure 4:**
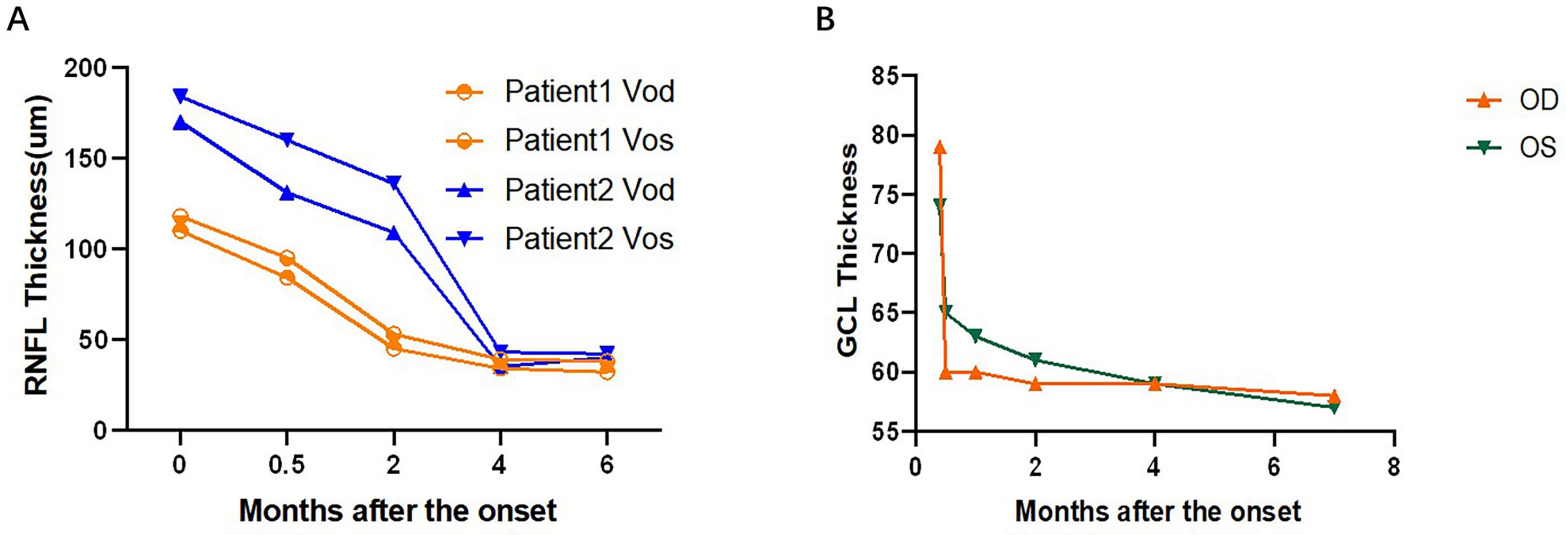
Line graph of the thickness of the development of RNFL and GCL in the course of the disease progression during the patient’s follow-up of 6 months. GCL, retinal ganglion cell layer.

Two patients (No.7 and No.11) had multiple RNFL measuring results. The trend of RNFL thickness changes is as follows (Figure 4A). The thickness of the RNFL decreased significantly after the onset of the first 2 months. Patient No.7 had multiple results from GCL measurement, and the trend of the statistical changes is as follows (Figure 4B), which showed that severe damage to the GCL occurred within the 1st month after the onset.

### 3.6. Changes in Retinal nerve fiber layer thickness over time and impact on visual acuity prognosis

The variation in RNFL thickness during the 1st month was selected for Pearson’s correlation analysis. The Pearson correlation coefficient was 0.18 (*p* = 0.001 and <0.05), indicating a positive correlation between VA and RNFL thickness. A mixed linear model analysis was performed including BCVA as a fixed effect variable and month and thickness as covariates. Month and thickness were statistically significant at the *α* = 0.05 level. The corresponding conditional mean prediction equation was BCVA (logMar) = 1.881166–0.160696*month+0.002042*RNFL thickness, from which the prognostic BCVA of the patients could be predicted based on the expected thickness of RNFL at different time points after the onset.

### 3.7. Correlation analysis of prognostic visual acuity with methanol poisoning

**Figure 5:**
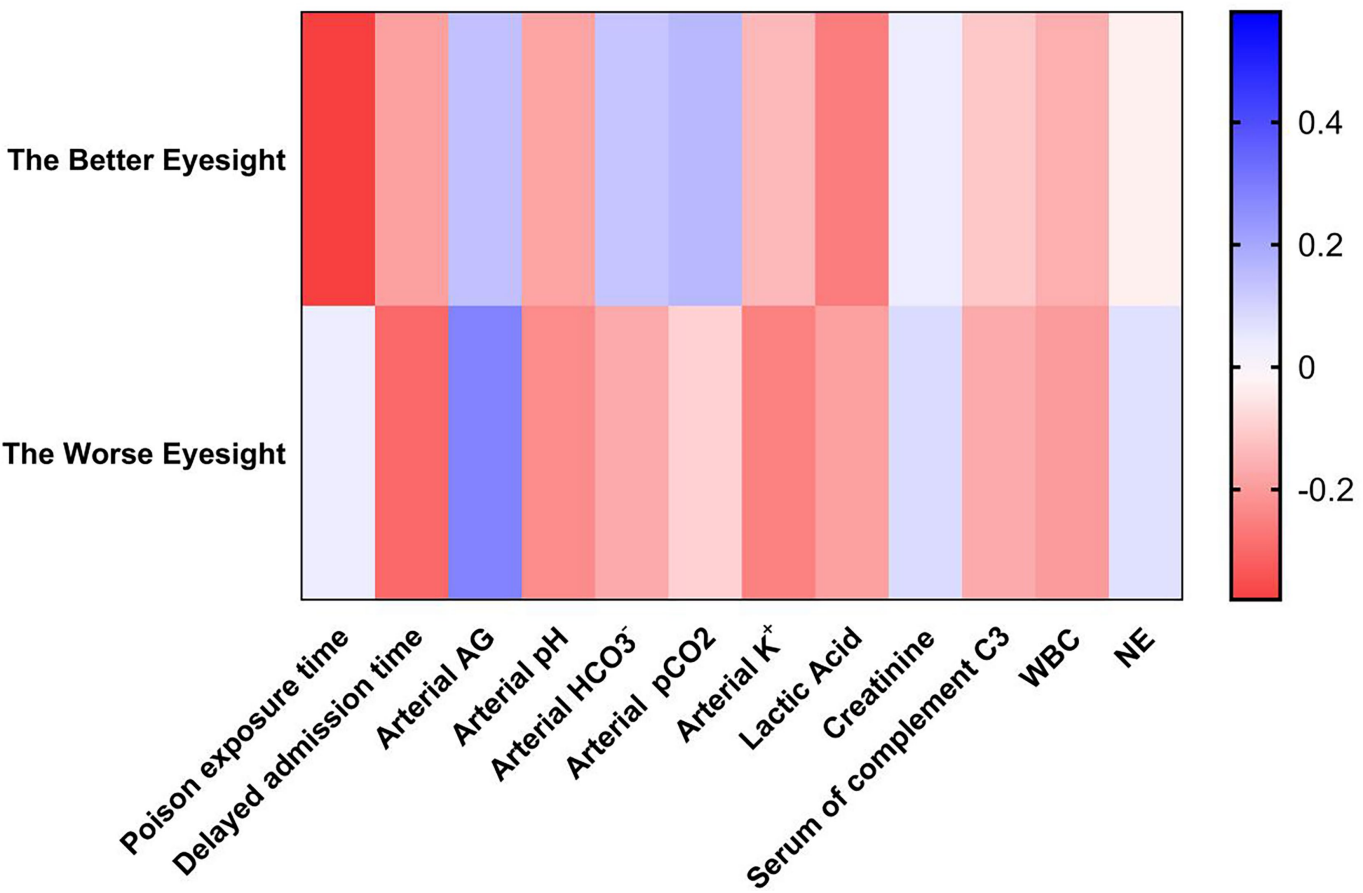
Heatmap of vision prognosis and risk analysis of inhalational methanol poisoning. The better eyesight was referred to as the better vision of the patient’s binocular vision. The worse eyesight was referred to as the worse vision of the patient’s binocular vision. AG, anion gap; WBC, white blood cell; NE, neutrophil.

Spearman’s analyses were used to analyze the correlation between the BCVA prognosis and various laboratory tests. The results showed that the factors that had a greater correlation with the better prognosis of BCVA were the time of exposure to toxicants (*r* = −0.38; *p* = 0.035) and blood lactate (*r* = −0.26; *p* = 0.043). The factors that had a greater correlation with the worse VA prognosis were delayed admission time (*r* = −0.3; *p* = 0.031) and arterial blood AG (*r* = 0.28; *p* = 0.035). The parameters with smaller correlations were arterial blood pH (*r* = −0.23; *p* = 0.046) and blood potassium concentration (*r* = −0.21; *p* = 0.050). Eyes with better VA prognosis were referred to as the better vision of the patient’s binocular vision. Eyes with worse VA prognosis were referred to as the worse vision of the patient’s binocular vision (Table 4; Figure 5).

**Table 4:** Spearman correlation analysis of VA prognosis and influencing factors.

Variable	Eyes with better VA prognosis		Eyes with worse VA prognosis	
	*p* value	*r*	*p* value	*r*
Exposure time to toxicants	0.035	−0.38	0.080	0.04
Delayed admission time	0.051	−0.19	0.031	−0.3
Arterial blood anion gap (AG)	0.060	0.14	0.035	0.28
Arterial blood acidity (pH)	0.053	−0.18	0.046	−0.23
Arterial blood bicarbonate HCO3-	0.062	0.13	0.063	−0.17
Arterial blood pCO2	0.056	0.16	0.074	−0.09
Blood K+	0.061	−0.14	0.050	−0.21
Lactic Acid	0.043	−0.26	0.054	−0.19
Creatinine	0.42	0.04	0.075	0.08
Serum complement c3	0.067	−0.11	0.063	−0.17
White blood cell (WBC)	0.056	−0.16	0.052	−0.2
Neutrophil (NE)	0.55	−0.03	0.077	0.07

### 3.8. Flash electroretinography

The six patients (12 eyes) with methanol poisoning in the acute phase at onset underwent ERG testing. All the latency and amplitude of waves a and b were in the normal range.

### 3.9. Pattern visual evoked potential

All nine patients (18 eyes) with methanol poisoning in the acute phase of the disease were given VEP tests, and seven eyes exhibited the disappearance of the P-VEP wave. The latency of the P_100_ wave was significantly prolonged in the remaining 11 eyes, and the amplitude of the P_100_ wave was significantly reduced.

### 3.10. Magnetic resonance imaging analysis of methanol poisoning

MRI was performed on all 14 patients with methanol poisoning, and only one patient was detected with no damaged lesion. The remaining 13 patients were found with varying brain damage and optic nerve injury at different sites.

Six patients presented with optic nerve impairment, of whom four showed thickening of the intraorbital segment of the optic nerve, two showed increased T_2_WI sequence signal of the optic nerve, and two showed increased DWI sequence signal of the optic nerve. In 13 patients, all had intracranial lesions, of which the most common manifestations were ischemic lesions in the basal ganglia and the frontal lobe in nine and three cases, respectively. In addition, there was an ischemic lesion in the cerebellar hemispheres, hippocampus, thalamus, parietal lobe, temporal lobe, insula, and hemi-oval region (Table 5).

**Table 5:** Analysis of MRI findings in the brains of patients in the acute stage of the disease.

Brain damage	Number of cases
Optic nerve thickening in the intraorbital segment	4
Optic nerve hyperintensities on DWI sequence	2
Optic nerve hyperintensities on T_2_WI sequence	2
Ischemia in the basal ganglia	9
Cerebellar hemisphere ischemia	1
Hippocampus ischemia	1
Thalamic ischemia	1
Frontal lobe ischemia	3
Parietal lobe ischemia	1
Temporal lobe ischemia	1
Insula ischemic	1
Hemianopia ischemia	1

MRI, magnetic resonance imaging; DWI, diffusion-weighted imaging; T_2_WI, T_2_ weighted image.

## 4. Discussion

This study was the first multifaceted case series study of inhalational methanol poisoning, with detailed ophthalmic and systemic medical material and at least 6 months of follow-up. It had been reported that 40% of patients with acute methanol intoxication had long-term visual sequelae, with 8% having blindness (6). Methanol poisoning could cause severe damage to visual function. For example, in this case series, the BCVA of 21 eyes declined to no light perception at the time of onset. However, 16 eyes showed different degrees of improvement, with the final BCVA (LogMar) ranging mainly from 2 to 3, and the vision stabilized after the 5th month.

With active treatment, visual function improved to varying degrees in all 14 cases in this study. The influencing factors for prognosis in inhalational methanol poisoning were exposure time (3), severity of metabolic acidosis, serum methanol concentrations, and ethanol concentrations at admission (4, 6). In this study, we found stronger predictors of VA prognosis, including longer exposure time to toxicants, delayed hospital admission, and lower arterial blood AG. Furthermore, elevated blood lactate, arterial blood acidity, and blood potassium concentration also affected the VA prognosis in some way. Of the 28 eyes we studied, nine eyes showed a trend of secondary VA decline between 0.5 and 2 months. We previously reported two patients (four eyes) with secondary visual loss after inhalational methanol poisoning (5).

**Figure 6:**
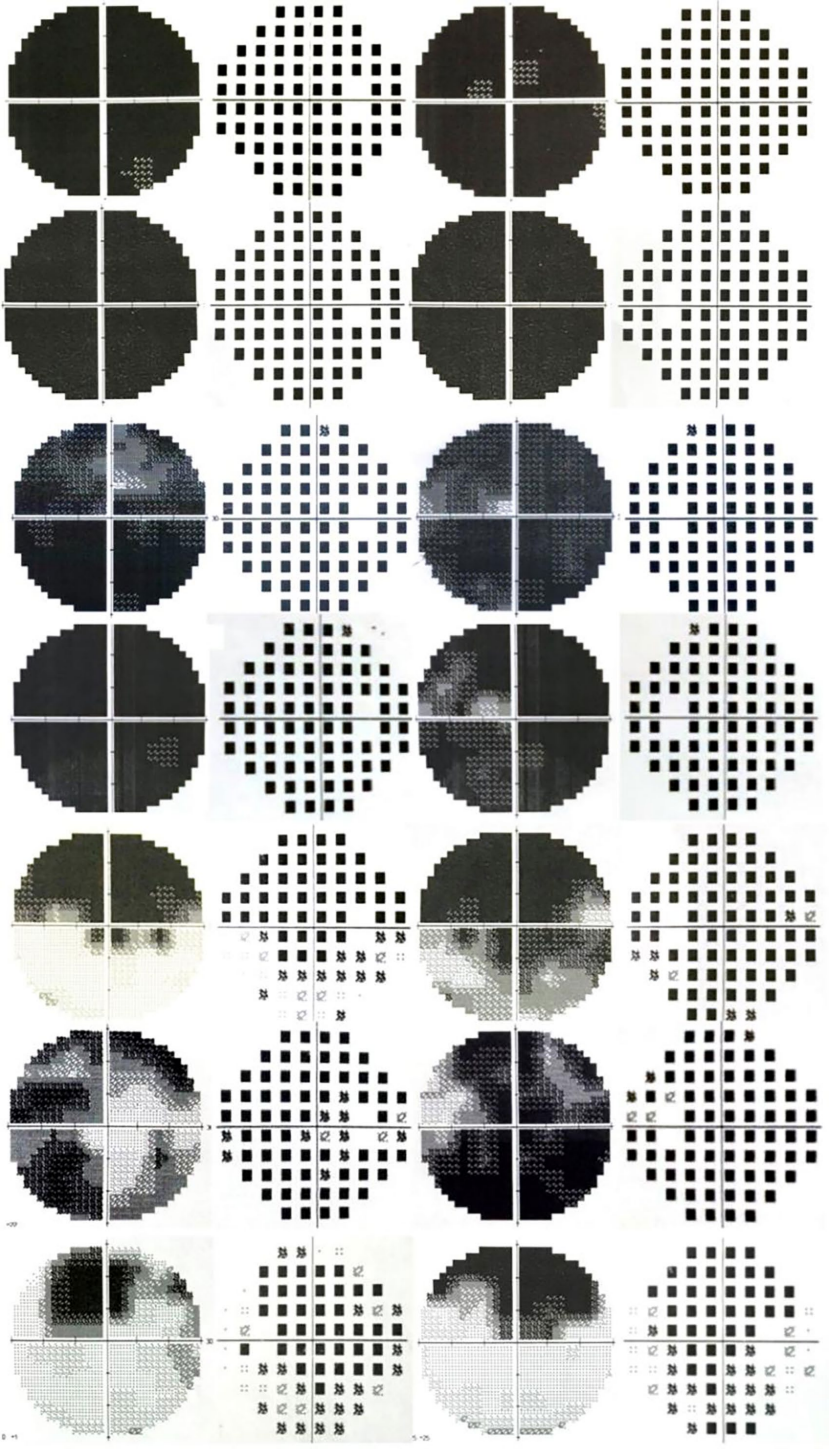
VF examination results during the acute phase of the disease.

As to the VF damage in patients with inhalational methanol poisoning, no literature has been reported. In this study, the VF pattern in the acute phase was mostly diffuse damage. Other patterns included total loss with relative preservation of the central VF (tubular VF), large paracentral scotomas, hemipleural VF defects, or temporal visual island (Figure 6). VF damage was previously found to be more severe in patients with acute eosinophilic poisoning of another origin other than methanol (7, 8).

Up to 24% of patients with acute methanol intoxication have been reported to have thinning of RNFL/GCL (9), suggesting damage to retinal ganglion cells and their axons. However, no studies have been reported on RNFL/GCL/OCTA after inhalational methanol poisoning. In our present study, early RNFL thickening due to the edema was very prominent (onset RNFL thickness 146.8 ± 33.2 μm), which subsided in the 1st month, showed dramatic thinning in the 3rd month, and then stabilized after the 5th month of the onset. The changes in RNFL thickness were followed by reductions of 44, 63, and 75% in the 3rd and 6th months, respectively, compared to the beginning. We included different variables in the mixed linear model and found that RNFL thickness was a more sensitive predictor of visual function. Therefore, VA can be predicted according to the prediction formula (as shown in the result). Only one patient had complete GCL data from 0 to 6 months, which showed very severe thinning within 1 month after poisoning. After 1 month, no change in GCL thickness was found, which might be due to complete injury to the papillomacular tract and its cell body. In terms of other patients, the GCL damage was also very severe. OCTA in two patients showed a substantial decrease in the density of the inner perioptic disk vessels and a mild decrease in the density of the outer vascular network. This phenomenon might be secondary atrophy of the inner vascular network due to RNFL atrophy.

In terms of electrophysiological changes following methanol intoxication, the literature reported prolonged latency of visual evoked potentials (VEP) due to axonal demyelination 1–9 months after acute methanol intoxication (10). The progressive decrease in P-wave amplitude on VEP examination over several years of follow-up represented the decrease of conductance due to the axonal remyelination, reflecting the characteristics of chronic axonal damage after methanol intoxication (11). This study found that most patients with inhalational methanol poisoning in the acute phase showed prolonged P100 wave latency and decreased amplitude, while some patients with no waveform indicated severe axonal damage. However, the ERG of the patients in this study was found normal, indicating that photoreceptor cells were waived of methanol poisoning. This might be because RGC cells are more dependent on the function of mitochondrial oxidative phosphorylation adenosine triphosphate (ATP) in energy supply than other cell components of the retina, and methanol might particularly seriously damage it.

The typical manifestations of MRI in acute methanol intoxication reported were necrosis of the bilateral basal ganglia, subcortical and deep white matter lesions, cerebral, cerebellar cortical lesions, midbrain lesions, and cerebral and ventricular hemorrhage, with even enhancement of necrotic lesions (12, 13). Our study found that the most common manifestations of brain MRI were ischemic necrosis in the basal ganglia and other ischemic changes in the frontal lobes. In addition, ischemic changes were also observed in the cerebellar hemispheres, hippocampus, semi-oval areas, thalamus, parietal lobe, temporal lobe, and insula. The involvement of the basal ganglia might be related to the direct toxic effect of methanol metabolites and the selective susceptibility of the basal ganglia to acidosis (14). Moreover, this study also found an abnormally enhanced signal in the optic nerve, mainly in the intra-orbital segment, mostly in the T2 and DWI sequences, providing evidence that the damage involved not only the optic disk but also other parts of the optic nerve as well. Similar findings in inhalational methanol intoxication have previously been reported, showing an enhanced signal of the optic nerve bilaterally and a signal restricting DWI of the posterior segment of the optic nerve bilaterally (14).

The limitations of the study were that the cases of inhalational methanol poisoning were extremely rare, and it is possible that the sample size was not large enough to achieve a positive conclusion in some way. There was a 14-year-old child among the subjects who violated the laws and the Declaration of Helsinki. We strongly call for the prohibition of child labor recruitment. Furthermore, because this was a retrospective study and some patients were transferred from other hospitals, there were some incomplete data, including toxicological tests, laboratory tests, and ocular examinations. However, despite these limitations, our study still presented several opinions of clinical significance. Inhalational methanol poisoning can lead to severe visual and optic nerve impairment in the optic disk and retrobulbar segments. The site of the CNS was the basal ganglia region. The main influencing factors include the duration of toxic exposure, delayed admission, and the degree of acidosis.

## Data availability statement

The original contributions presented in the study are included in the article/supplementary material, further inquiries can be directed to the corresponding author.

## Ethics statement

The studies involving human participants were reviewed and approved by Zhongshan Ophthalmic Center’s Ethics Review Committee. Written informed consent to participate in this study was provided by the participants’ legal guardian/next of kin. Written informed consent was obtained from the individual(s) for the publication of any potentially identifiable images or data included in this article.

## Author contributions

HY provided guidance and direction throughout the writing and revision. HS handled the literature review, study design, data analysis and interpretation, and manuscript writing. LZ provided expertise in writing ideas. YF was responsible for the revision of the full text. WS was responsible for data collection and organization. YY undertook the translation task. Others assisted in related work. All authors contributed to the article and approved the submitted version.

## Funding

This study was financially supported by the grants from National Natural Science Foundation of China to HY (Grant Number: 81870656).

## Conflict of interest

The authors declare that the research was conducted in the absence of any commercial or financial relationships that could be construed as a potential conflict of interest.

## Publisher’s note

All claims expressed in this article are solely those of the authors and do not necessarily represent those of their affiliated organizations, or those of the publisher, the editors and the reviewers. Any product that may be evaluated in this article, or claim that may be made by its manufacturer, is not guaranteed or endorsed by the publisher.
